# Exploring the role of *RALYL* in Alzheimer’s disease reserve by network-based approaches

**DOI:** 10.1186/s13195-020-00733-z

**Published:** 2020-12-09

**Authors:** Yixuan Zhang, Jiali Wang, Xiaoquan Liu, Haochen Liu

**Affiliations:** 1 School of Pharmacy, China Pharmaceutical University, Nanjing, 210009 People’s Republic of China; 2 Center of Drug Metabolism and Pharmacokinetics, China Pharmaceutical University, Nanjing, 210009 People’s Republic of China

**Keywords:** *RALYL*, AD reserve, Expression dynamics, Cognitive decline

## Abstract

**Background:**

Alzheimer’s disease (AD) reserve theory is based on specific individual characteristics that are associated with a higher resilience against neurodegeneration and its symptoms. A given degree of AD pathology may contribute to varying cognitive decline levels in different individuals. Although this phenomenon is attributed to reserve, the biological mechanisms that underpin it remain elusive, which restricts translational medicine research and treatment strategy development.

**Methods:**

Network-based approaches were integrated to identify AD reserve related genes. Then, AD brain transcriptomics data were clustered into co-expression modules, and a Bayesian network was developed using these modules plus AD reserve related phenotypes. The directed acyclic graph suggested that the module was strongly associated with AD reserve. The hub gene of the module of interest was filtered using the topological method. Validation was performed in the multi-AD brain transcriptomic dataset.

**Results:**

We revealed that the *RALYL* (RALY RNA Binding Protein-like) is the hub gene of the module which was highly associated with AD reserve related phenotypes. Pseudo-time projections of *RALYL* revealed the changes in relative expression drivers in the AD and control subjects over pseudo-time had distinct transcriptional states. Notably, the expression of *RALYL* decreased with the gradual progression of AD, and this corresponded to MMSE decline. Subjects with AD reserve exhibited significantly higher *RALYL* expression than those without AD reserve.

**Conclusion:**

The present study suggests that *RALYL* may be associated with AD reserve, and it provides novel insights into the mechanisms of AD reserve and highlights the potential role of *RALYL* in this process.

## Background

Alzheimer’s disease (AD) reserve refers to the differences in cognitive processes as a function of AD risk factors (genes, personality, lifestyle, and external environment) that explain differential susceptibility to functional impairment during pathology or other neurological insults [1–3]. AD risk factors or pathology increases at the same rate in two individuals with a high and low reserve. The number of risk factors or pathology needed before the cognitive function is greatly affected by a higher reserve, leading to a later change point of cognitive decline. More pathology will be needed for an individual with a higher reserve to meet clinical diagnostic criteria for AD, thus delaying the onset of the disease [2, 4]. There is no evolutionary pressure in creating systems hat actively protect the brain from any aging pathology, let alone systems that offer protection from different pathologies [5]. However, since all human physiologic systems exhibit a reserve, a hypothetical therapeutic strategy could be used to offset nearly all common AD brain pathologies that alter cognition.

Regarding AD cognitive performance, AD reserve refers to the ability to maintain cognitive function despite the accumulation of AD pathologies that contribute to cognitive decline. Increasing numbers of clinical studies show that, in a large proportion of normal aging, no individuals with cognitive impairment have sufficient numbers of plaques and tangles to meet the neuropathologic criteria for AD, and they do not manifest dementia symptoms during their lifespan [6]. For instance, the first report by Blessed et al. in 1968 revealed that 6 subjects had no dementia but exhibited a high amyloid-β count [7]. In another ROSMAP study, individual trajectories of cognitive decline were calculated from longitudinal cognitive measures that include up to 20 yearly evaluations. It was revealed that 46% of the participants had pathologic AD but without clinical dementia [8]. Continuous evidence from epidemiology, imageology, and neuropsychology suggests that higher reserve significantly delays the onset of dementia in early AD and suppresses the rate of cognitive decline in AD advanced stages [9–12]. Given the high complexity and multifactorial etiology in AD, a method that incorporates the latest evidence from related disciplines is promising in the study of AD reserve. The reserve is a heuristic to aid in elucidating individual differences in cognition, function, or clinical status relative to AD and explore potential therapeutic strategies. However, the biological mechanisms that underpin the protective effects have not been fully elucidated, limiting the development of more effective preventive and treatment strategies.

Many novel potential AD therapeutic targets have been identified using transcriptomics, proteomics, and metabolomics [13]. However, it is difficult to transition these single isolated molecular targets to a complete mechanism that causes cognitive decline, characteristic accumulation of amyloid-β and neurofibrillary tangle pathology, or the AD reserve. A network-based perspective provides a more nuanced molecular definition of AD than a simple single-gene association by developing a systematic framework with which to assemble disparate single-gene findings into disease mechanisms [8, 14, 15]. Network or the graph theory approaches can joint modeling neuropathologic burden, and cognition performance with the target gene has better performance in capturing the relationship between molecular and different stages of the AD reserve process to clarify the potential underlying mechanisms.

We used the gene module to the phenotype network (MPN) approach to evaluate AD reserve related genes that impact cognitive function and pathological change. A gene module is defined as a cluster of genes with similar expression profiles in a physiological process. MPN aids in constructing gene modules and identifies those that are directly associated with cognitive decline, conditioned on neuropathology, and other large-scale transcriptomic changes in the human AD brain. The aim of MPN is to evaluate beyond single-gene level associations when defining robust molecular mechanisms while avoiding the limitations of pathways derived from single databases. Modeling transcriptome changes into gene modules prioritizes specific highly connected genes (hub genes) within a module for further research. Targeting and rebuilding such hub genes to homeostasis have been proposed as a pathway for disease treatment [16, 17].

**Fig. 1:**
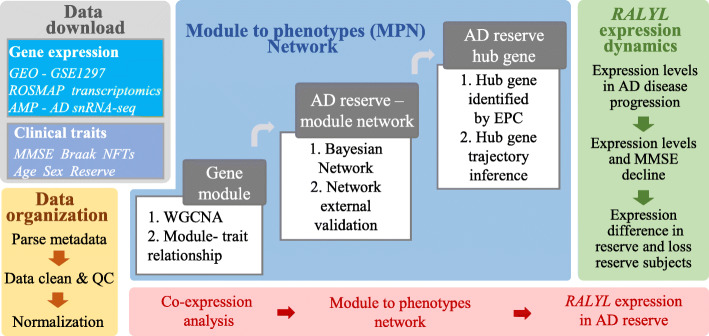
Pipeline of the MPN to prioritize modules and genes directly associated with AD reserve in our study. Gray and yellow block: data download and organization. Blue block: MPN modeling. Gene module: applied WGCNA to GSE1297 microarray expression data to identify co-expression modules and calculate the correlation between modules and each phenotype. AD reserve-module network, modules, and phenotypes are combined using conditional independence relationships (via Bayesian networks) to identify direct relationships among gene modules, AD traits, and reserve. The module most strongly associated with reserve is the target module and independently validated in the ROSMAP expression data. AD reserve hub gene: exploring the AD reserve hub genes in the target module and trajectory inference. Green block: *RALYL* expression validated in multi-AD RNA-seq data

In this study, an integrative research strategy was utilized in three parts. First, weighted gene co-expression network analysis (WGCNA) was performed to cluster AD and differentially expressed genes into gene modules for the construction of MPN. Second, a Bayesian network integrating gene modules and reserve phenotypes was used as a directed acyclic graph (DAG) to identify the gene module that is most strongly associated with AD reserve. Third, the AD reserve correlated gene was selected from module hub genes and validated in the multi-transcriptomics dataset which included the single-nucleus RNA (snRNA-seq) sequencing dataset. Exploring the relationship between gene expression dynamic and cognitive changes in Alzheimer’s disease, from a reserve perspective, provides insights into the prevention and treatment of AD. The research strategy of this study was as presented in Fig. 1.

## Materials and methods

### Data origin and acquisition

The microarray gene expression data used in constructing gene modules and validation were obtained from the NCBI GEO database (GSE1297 and GSE28146). Hippocampal gene expression of AD human subjects and normal aging controls of varying severity including 7 incipient, 8 moderate, and 7 severe cases were analyzed. Clinical trait metadata included hippocampal neurofibrillary tangles (NFTs), Braak staging, Mini-Mental State Examination (MMSE), sex, age, and postmortem interval (PMI) [18].

The module-phenotype network external validation data was acquired from two prospective clinical-pathologic cohort studies on aging and dementia: the Religious Orders Study (ROS) and the Memory and Aging Project (MAP). The two studies (collectively referred to as ROSMAP) share clinical and neuropathological properties, allowing data pooling. We used ROSMAP microarray expression and RNA-seq data for this work.

All microarray and bulk RNA-seq raw data for gene expression were normalized by *z*-score normalization; a *z*-score describes the position of a raw score in terms of its distance from the mean, when measured in standard deviation units; the *z*-score is positive if the value lies above the mean and negative if it lies below the mean. It is useful to standardize the gene expression matrix of a normal distribution by converting them into *z*-scores because it allows to calculate the probability of a score occurring within a standard normal distribution and compares two scores that are from different samples (which may have different means and standard deviations).

The snRNA-seq data was downloaded from the Single-cell atlas of the Entorhinal Cortex in the Human Alzheimer’s Disease database [19]. The DroNc-Seq data on the 10X platform was obtained from the entorhinal cortex of 6 Alzheimer’s disease patients as well as from 6 sex- and age-matched controls. Using the Grch38 (1.2.0) reference from 10X Genomics, we established a pre-mRNA reference according to the detailed steps using 10X Genomics. Cell ranger count was used to obtain raw counts. To disentangle the individual donor identity of every cell, we used the Bayesian demultiplexing tool vireo (Version 0.1.2) and its associated pipeline.

### Construction of WGCNA and identification of modules

Weighted correlation network analysis (WGCNA) was used to identify clusters (gene modules) of highly correlated genes. These clusters were summarized using the module eigengene or an intramodular high connected gene to identify related modules and external sample traits, as well as to calculate measurements for module membership. Following the WGCNA framework, we modeled WGCNA using R (3.6.1) based on the microarray data (GSE1297). The Pearson correlation coefficient was used to determine similarities in gene expression profiles. The correlation coefficients between genes weighting by a power function were obtained through a scale-free network. In terms of co-expression, gene modules were considered to be densely interconnected gene clusters.

WGCNA uses hierarchical clustering to identify genetic modules. We used a hierarchical clustering dendrogram for visualization. Modules in the dendrogram were indicated by different colors. Dynamic tree cutting methods were used to distinguish between different modules. During module selection, the adjacency matrix was used to measure topological similarities that were converted to a topology overlay matrix (TOM). Then, modules were detected through cluster analysis [20]. The biological coherence of these modules was validated from three perspectives: (i) the associations of the module eigengene (ME, the first principal component of the module, represented the global expression level of the module) to phenotypes obtained from Pearson’s correlation analysis, (ii) the correlation coefficient between module membership (gene expression levels) with gene significance (GS, for assessing the association between genes and phenotypes) was calculated and the *p* values obtained, and (iii) Gene Ontology (GO) [21] functional enrichment analysis.

### Bayesian network structure learning to modeling the module-phenotype network

A Bayesian network is a probability graphical model that represents a set of random variables and their conditional dependencies using a directed acyclic graph (DAG). Bayesian structure learning algorithms (such as the Hill-Climbing algorithm used in this work) enable the estimation of the DAG, describing the conditional dependencies between a set of random variables in a data-driven manner. We used R (3.6.1) package bnlearn modeling Bayesian network to determine the DAG that describes the relationship between three types of variables: gene modules, whether there is a reserve, and three main AD traits (Braak staging, NFT counts, and MMSE score) [22]. Joint gene modules and phenotypic traits were used to identify which gene module was strongly associated with reserve trait in DAG.

The classical ensemble Hill-Climbing approach used in this study was recently shown to be integrated into Bayesian network structure learning when used to analyze gene expression data. Structural learning was performed for a total of 200 times with stochastic initialization so that learning was not trapped in a local optimum. The Bayesian model averaging was used to select the final Bayesian network.

### Identification of the hub genes in module 11

The DAG obtained from the Bayesian network structure learning describes the conditional dependencies between a set of random variables. Thus, we identified the module that was strongly associated with cognitive decline and reserve phenotypes by analyzing the main parameters of the DAG nodes. The Markov blanket of a node is the set of nodes consisting of its parent nodes, its children nodes, and any other parent nodes of its children nodes; the Markov blanket renders the node independent of the rest of the DAG. The Markov blanket for one module is the best-selected subset of the Bayesian network for that module and represents the characters of the module; if the Markov blanket of module A does not contain module B or phenotype X, we can assume that module A is conditionally independent of module B and the phenotype X. The association between gene module and reserve was identified through the Markov blanket. The more AD trait was comprised in the Markov blanket of a module, the more strongly it was associated with these traits.

The Bayesian network structure learning framework described above identified module 11 (M11) as being the most strongly associated with cognitive decline and reserve phenotypes. Therefore, we devised another Bayesian network to prioritize genes in the target module (which contained 262 genes) for further validation. Genes in the target module were screened according to the following two considerations: (i) There must have been no latent variables (biological meaning still not clear genes cannot be used as variables in the network) acting as confounding factors. Such variables induce false correlations between the observed variables, thus introducing bias in the causal network. (ii) Smaller Bayesian networks were several non-equivalent networks that equally fit the data and could be used to identify a set of likely causal networks that fit our biological knowledge.

Next, we constructed a Bayesian network to represent conditional dependencies between genes in the module that were strongly associated with the reserve trait in a DAG. We used Cytoscape plugin cytoHubba for ranking nodes in the DAG by utilizing their network features [23]. The hub genes and their subnetworks were obtained by topological analysis algorithm edge percolated component (EPC). Global-based categorization method based on percolation in random graphs was used to compute how strongly a protein associates with the other parts of the network and how significantly an interaction contributes to the integrity of the network [24].

### AD reserve hub gene validation and snRNA-seq developmental trajectory analysis

Processed snRNA-seq data (the expression data and sample metadata) was downloaded, and the standard quality control pre-processing workflow performed using Seurat 3.1 [23]. Cells with unique feature counts over 2500 or less than 200 and cells with > 5% mitochondrial counts were filtered. After the elimination of unwanted cells from the dataset, a global-scaling normalization method “LogNormalize” was used to normalize the feature expression measurements for each cell by the total expression multiples, using a scale factor and log-transforms of the result. Major cell types of the human brain were identified and annotated by interrogating the expression patterns of known marker genes [19]: neurons (marked by *GRIK2*, *GRIA1*, *GRIN2B*, and *RBFOX1*), astrocytes (*AQP4* and *SLC1A2*), oligodendrocytes (*MBP*, *MOBP*, and *PLP1*), microglia (*HLA-DRA*, *CX3CR1*, *C1QB*, and *CSF1R*), and oligodendrocyte progenitor cells (*PCDH15* and *MEGF11*). If the above markers were found to not exist in one cluster, or if there were multiple cell markers in one cluster at the same time, then, the cluster was classified as “unidentified.” Single-cell developmental trajectories along pseudo-time were performed by Monocle 2, an R package that orders single-cell transcriptomes and uses machine learning in an unsupervised manner for reconstruction [24, 25]. It reveals both known and novel genes that are expressed along a developmental trajectory [26].

The hub gene for the AD reserve was identified using the above approach. To study the dynamic characteristics of the hub gene in the AD disease progression, the expression levels of the hub gene were determined based on the clinical diagnoses of patients in GSE1297, GSE28146, and ROSMAP AD RNA-seq dataset groups. Expression levels were determined in different groups, including different AD disease severity (normal aging, incipient AD, moderated AD, and severe AD), different disease stages (no cognitively impaired (NCI), mild cognitive impairment (MCI), AD dementia), and subjects with reserve or loss reserve. MMSE score decline during clinical follow-up between high and low hub gene expression subjects was also calculated.

### Statistical analysis

All statistical data were analyzed and visualized using the ggplot2 and ggpubr (ggplot2 Based Publication Ready Plots) of R (3.6.1). Means were compared using a *t* test or one-way ANOVA. Multiple testing of *p* value was performed using the Bonferroni method, and adjusted *p* value as reported. The acceptable level of significance was established at *p* ≤ 0.05.

## Results

### Data cleaning and pre-processing

Procedures for cleaning data (GSE1297 dataset) obtained from the GEO database and used in constructing the gene module were as shown in Fig. 1. Using this dataset, gene expression of human hippocampus on 31 separate microarrays, primarily based on MMSE criteria, was analyzed. Subjects were initially categorized into four groups [18]: 9 controls (MMSE > 25), 7 incipient AD (MMSE 20–25), 8 moderate AD (MMSE 14–19), and 7 severe AD (MMSE < 14). Based on the concept of AD reserve [2, 27], subjects in GSE1297 were initially categorized into two groups, termed “reserve” (MMSE > 26, Braak ≥ 3), and “loss reserve” (MMSE < 26). Several borderline cases (e.g., MMSE = 26) were assigned based on NFT, β-amyloid plaque, and Braak stage data. Therefore, 5 subjects were allocated in AD reserve, while 26 subjects were allocated in loss AD reserve.

**Fig. 2:**
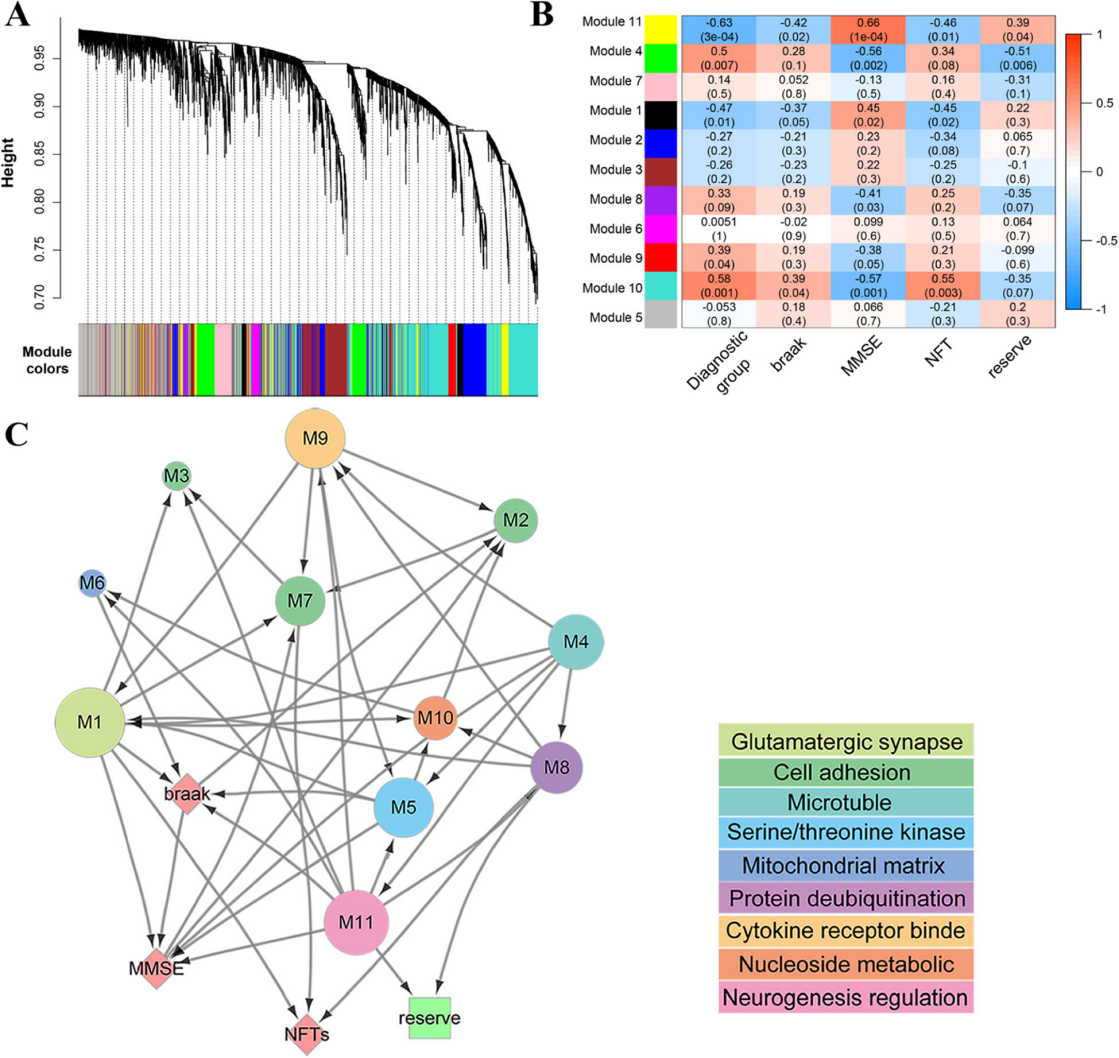
Module to phenotype network (MPN). **a** WGCNA hierarchical clustering dendrograms of identified eleven distinct modules of highly co-expressed genes; all modules are marked with different colors. **b** The correlation between modules and phenotypes. Heatmaps of the correlation between eigengene and AD phenotypes (covariates: age, sex, PMI), each cell contains the corresponding correlation and *p* value, and the correlations between module 11 and AD phenotypes: diagnostic group (cor = − 0.63, adjusted *p* = 0.4 × 10^−3^), reserve (cor = 0.39, adjusted *p* = 0.04), MMSE (cor = 0.68, adjusted *p* = 0.1 × 10^−3^), and Braak (cor = − 0.42, adjusted *p* = 0.02). **c** A directed acyclic graph, obtained using Bayesian network structure learning, represents the relationships between modules (circles), three relevant AD phenotypes (NFTs, Braak scores, and cognitive performance (MMSE); diamonds), and AD reserve (squares). The diameter of the circles is proportional to the number of genes in the module. The color of the module corresponds to the legend and represents the results of Gene Ontology (GO) analysis of each module

All 31 GSE1297 subjects were evaluated using the hierarchical clustering method (Supplements Fig. 2a). The raw data for gene expression was normalized by *z*-scores to obtain the expression matrix. Probes without corresponding annotation information were eliminated, and the standard deviation of all gene expressions was computed to obtain a list sorted by decreasing standard deviations. Eventually, the top 25% of genes with large standard deviations were obtained. After data cleaning and pre-processing, there were 22,283 gene symbols in the dataset, and the rest of the 3100 genes were subjected to further modeling.

In ROSMAP microarray gene expression, there were 151 AD and no other cause of cognitively impaired participants, 57 MCI and no other cause of cognitively impaired participants, and 67 no cognitively impaired (NCI) as assessed by the clinical consensus diagnosis of ROSMAP clinical codebook that cut off criteria of AD from physician’s overall cognitive diagnostic results. At the time of death, all available clinical data were reviewed by a neurologist with expertise in dementia, and a summary diagnostic opinion regarding the most likely clinical diagnosis at the time of death was provided. The summary of diagnoses was blinded to all postmortem data. Case conferences including one or more neurologists and a neuropsychologist gave a consensus on selected cases. According to the AD reserve criterion (see above), there were 37 subjects with AD reserve, whereas 70 subjects with lost AD reserve. A follow-up on 145 subjects (both had the first diagnostic MMSE score and last valid MMSE score) was done to assess the correlation between cognitive decline and target module.

### Constructing gene module-phenotype network

To model large-scale transcriptome changes into gene modules, we identified groups of co-expressed genes or gene modules. Gene modules represented transcriptional regulatory mechanisms, including transcription factors, chromatin conformation, and related underlying factors, such as the proportion of different cell types in the sample tissue. In developing the gene module, we applied the WGCNA algorithm to GSE1297 microarray data which built a scale-free co-expression network; the scale independence and mean connectivity analysis demonstrated that when the soft threshold of scale-free co-expression network was equal to 6, the average degree of connectivity was close to 0, and the scale independence was more significant above 0.9 (Supplementary Fig. 1B), it means 6 is an appropriate soft threshold. By calculating the correlation coefficient between genes, we classified the genes with the same expression patterns into the same module. A total of 11 modules were identified, ranging between 38 and 853 gene members (assigning each module a color for reference, Fig. 2a) in terms of size.

Pearson’s correlation coefficient between the 11 modules and main AD phenotype (diagnostic group, Braak, MMSE, NFTs, and reserve) was calculated to identify modules that were significantly associated with clinical phenotypes. The influence of covariates (age, sex, and PMI) was considered in this calculation. The highest association in the module trait relationship was found between module 11 (yellow module) and clinical features including diagnostic group (cor = − 0.63, *p* = 0.3 × 10^−3^), MMSE (cor = 0.66, *p* = 0.1 × 10^−3^), Braak (cor = − 0.42, *p* = 0.02), and NFTs (cor = − 0.46, *p* = 0.01) (Fig. 2b). The expression level of module 11 was positively correlated with factors that maintain cognitive performance, but negatively correlated with AD pathology. We selected 4 AD phenotypes (reserve, MMSE score, Braak score, NFTs) and all the 11 gene modules for further modeling.

### Identifying modules associated with AD reserve in gene module-phenotype network

To separate a small number of direct module-phenotype associations from a large number of indirect module-trait correlations, we used Bayesian network inference [8] where random variables represented module gene expression levels and phenotype values. Edges with the arrow in Bayesian network represented direct conditional dependencies between variables: an arrow from node A to node B in a Bayesian network indicated that a value taken by variable node B was dependent on the value taken by variable node A, conditioned on all the other variables in the network (see the “Materials and methods” section). The resulting Bayesian network consisted of 43 edges and 15 nodes; 11 nodes represented gene modules and 4 AD phenotype nodes (Fig. 2c). All the 11 modules were enriched using the Gene Ontology (GO) analysis (Fig. 2c colored bar).

By analyzing the structure (Fig. 2c) and main parameters (Supplementary Table 1) of DAG from the Bayesian network, the Markov blanket of 4 modules (M6, M7, M8, and M11) contains reserve, indicating the best-selected subset of these 4 modules in Bayesian network contains reserve and the remaining modules were conditionally independent of the reserve. Of the 4 modules, only M8 and M11 were directly correlated to reserve as children nodes. Furthermore, relative to M8, MMSE is a children node of M11, indicating the dependency between M11 and MMSE. Through GO analysis of M11, the main enrichment results of the biological processes in this module were associated with positive regulation of neurogenesis, and cellular component was associated with neuronal cell body while molecular function was associated with cell adhesion molecule binding. Genes in M11 were more enriched in the nervous system than in M8. In summary, based on integrated mathematical and biological evidence, module 11 (M11) was strongly associated with cognitive decline (module MMSE) and AD reserve (module reserve), conditioned on all other correlated modules and those that represent AD phenotype.

**Fig. 3:**
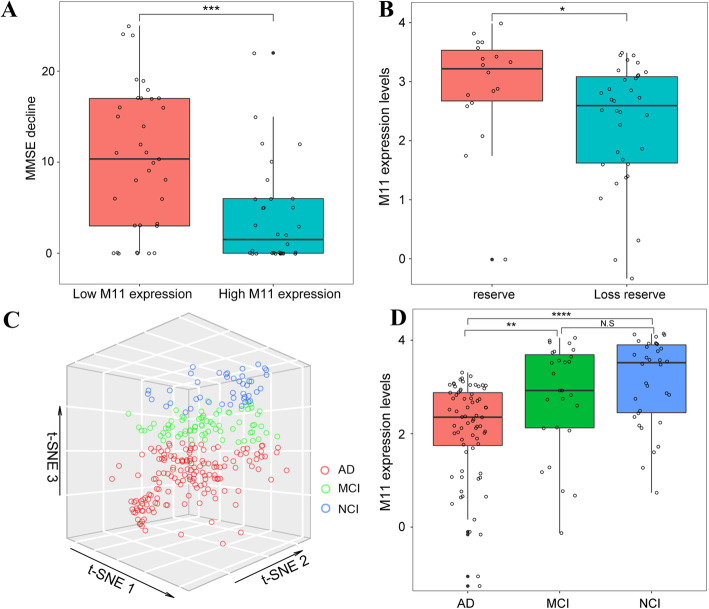
The MPN prioritizes module 11 as being directly associated with AD reserve. **a** Trajectories of cognitive decline for people with low (left) or high (right) levels of module 11 expression; low module 11 expression subjects have a steep cognitive decline than high module 11 expression (multiple *t* test, adjusted *p* = 0.00019). Red: MMSE score decline from the first AD diagnoses to last validation from subjects with low module 11 levels (fourth quartile of expression levels). Blue: MMSE score decline from the first AD diagnoses to last validation from subjects with high module 11 levels (first quartile of expression levels). **b** Expression of module 11 for individuals with reserve (red) and loss reserve (blue); each point represents one individual (multiple *t* test, adjusted *p* = 0.031). **c** NCI, MCI, and AD have a distinct module 11 gene expression characteristics. Each subject’s 262 genes in module 11 dimensionality reduction to three principal components using the t-SNE algorithm. One point represents one subject: three groups of subjects have distinct spatial division suggesting that module 11 genes partly represent the difference between NCI, MCI, and AD. **d** Expression of module 11 for individuals with AD (red), MCI (green), and NCI (blue); each point represents one individual (multiple one-way ANOVA, adjusted *p* = 0.72 × 10^−5^)

The association between module 11 with cognitive decline and AD reserve was externally validated in the independently processed ROSMAP microarray expression data. The trajectories of cognitive decline for people with low or high levels of module 11 expression are illustrated in Fig. 3a. Individuals with low module 11 expression exhibited a significant MMSE decline (multiple *t* tests, adjusted *p* = 0.00019). Besides, we compared expression levels between individuals with AD reserve and loss reserve (multiple *t* tests, adjusted *p* = 0.031) (Fig. 3b), and found that high module 11 expression is a potential reserve factor.

To evaluate the expression dynamics of module 11 in whole AD disease progression, individuals with NCI, MCI, and AD diagnosis were assessed by the dimensionality reduction algorithm t-SNE [28]. The dimension of all 262 genes in module 11 was significantly reduced in three dimensions; thus, we could visualize different AD stage subjects’ module 11 expression in a 3D image (Fig. 3c). The image indicated that NCI AD, MCI, and subjects exhibited independent expression characteristics and were well divided into dimension-reduced space. In module 11, the expression levels of NCI, MCI, and AD are depicted in Fig. 3d. A significant fall from MCI to AD stage indicated that the transcriptional alterations of module 11 mainly occur during the conversion of MCI to AD (multiple one-way ANOVA, adjusted *p* = 0.72 × 10^−5^).

### Hub gene in module 11 and trajectory inference in AD

The Bayesian network structure learning framework described above identified module 11 (M11) as the most strongly associated with cognitive decline and reserve phenotypes. Therefore, we devised another Bayesian network to prioritize genes in the target module (which contains 262 genes) for further validation. The Bayesian network is a probability graphical model that represents a set of genes and their conditional dependencies.

**Fig. 4:**
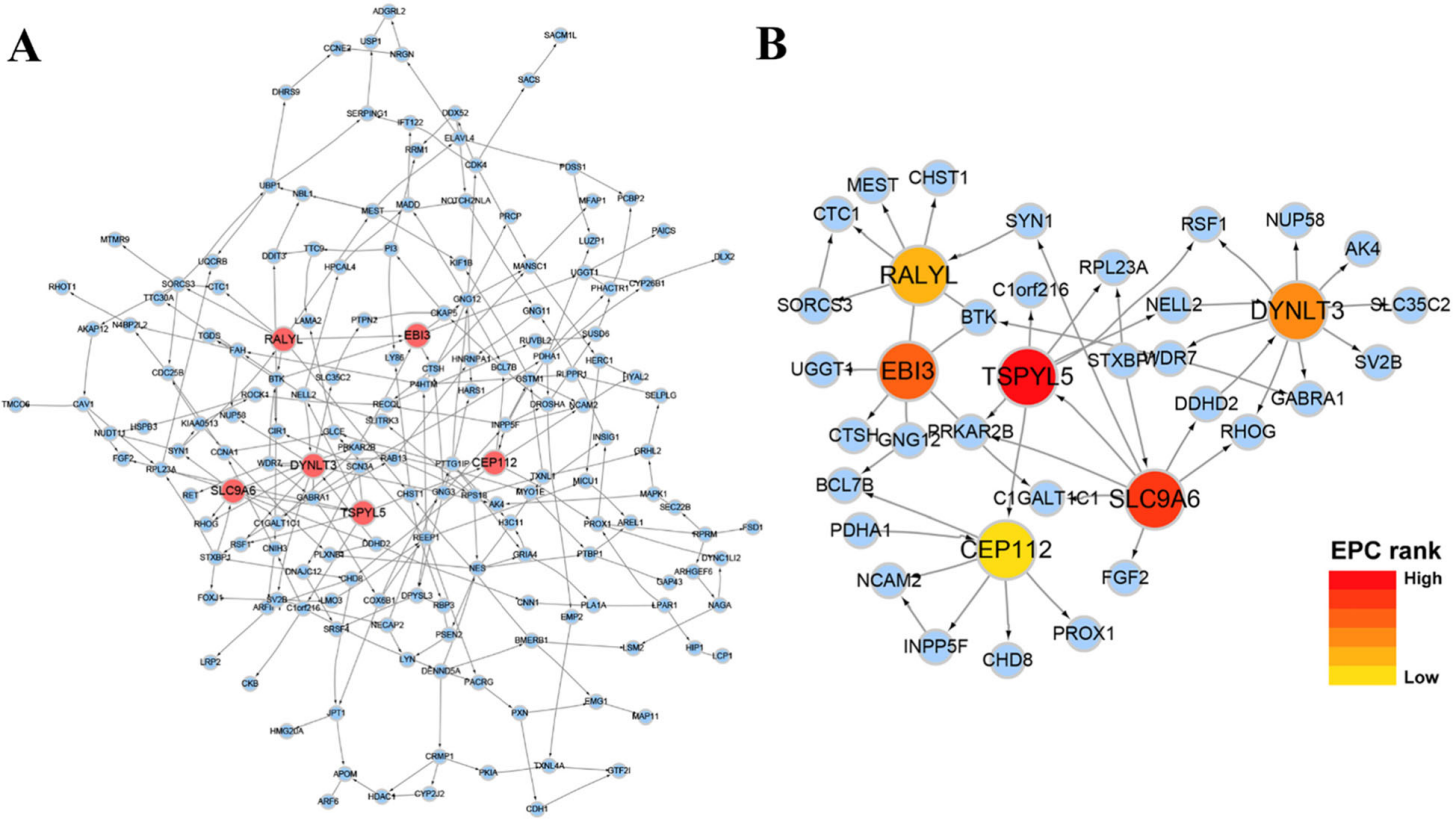
Identification of specific genes within module 11. **a** The estimated gene regulatory network (Bayesian network) for 169 selected genes in module 11. Each gene is a node (circle) in the displayed graph. Colored nodes are hub genes identified using edge percolated component (EPC) algorithm. **b** Hub gene and its expanded subnetwork; the transition of the module color from red to yellow represents a decrease in the EPC rank (the centrality of the module decreases from red to yellow)

First, the number of plausible genes was reduced and genes that were not associated with AD and cognition were excluded to ensure that the conditional dependencies in the network were of a considerable biological significance. Plausible genes were reduced by taking the top 169 genes (among the 262 genes assigned to target module) that were (i) associated with AD and cognitive performance, or played some role in the nervous system, and (ii) connected to other genes in an initial Bayesian network of 262 genes. Next, we constructed a Bayesian network for representing conditional dependencies between these 169 genes in a DAG (Fig. 4a).

Hub genes in DAG were identified through network topological analysis, considering the phenomenon that local centrality measure algorithms do not take into account the rest of the network. The importance attributed to its value strongly depended on the network size. Global centrality measures that accounted for the whole of the network and calculated the shortest paths between all nodes were chosen. Each node was then assigned a score based on the sum of the shortest paths to find the nodes that were best placed to rapidly influence the entire network. Edge percolated component (EPC) is one of the commonly used global centrality measures and has been found to have a good performance in finding the essential proteins in the protein interaction network of yeast [29]. Using EPC, we identified six hub genes within module 11: *SLC9A6*, *TSPYL5*, *EBI3*, *DYNLT3*, *RALYL*, and *CEP112*; the number of their neighbors’ nodes that were directly connected was 8, 7, 6, 10, 7, and 7, respectively (Fig. 4b).

**Fig. 5:**
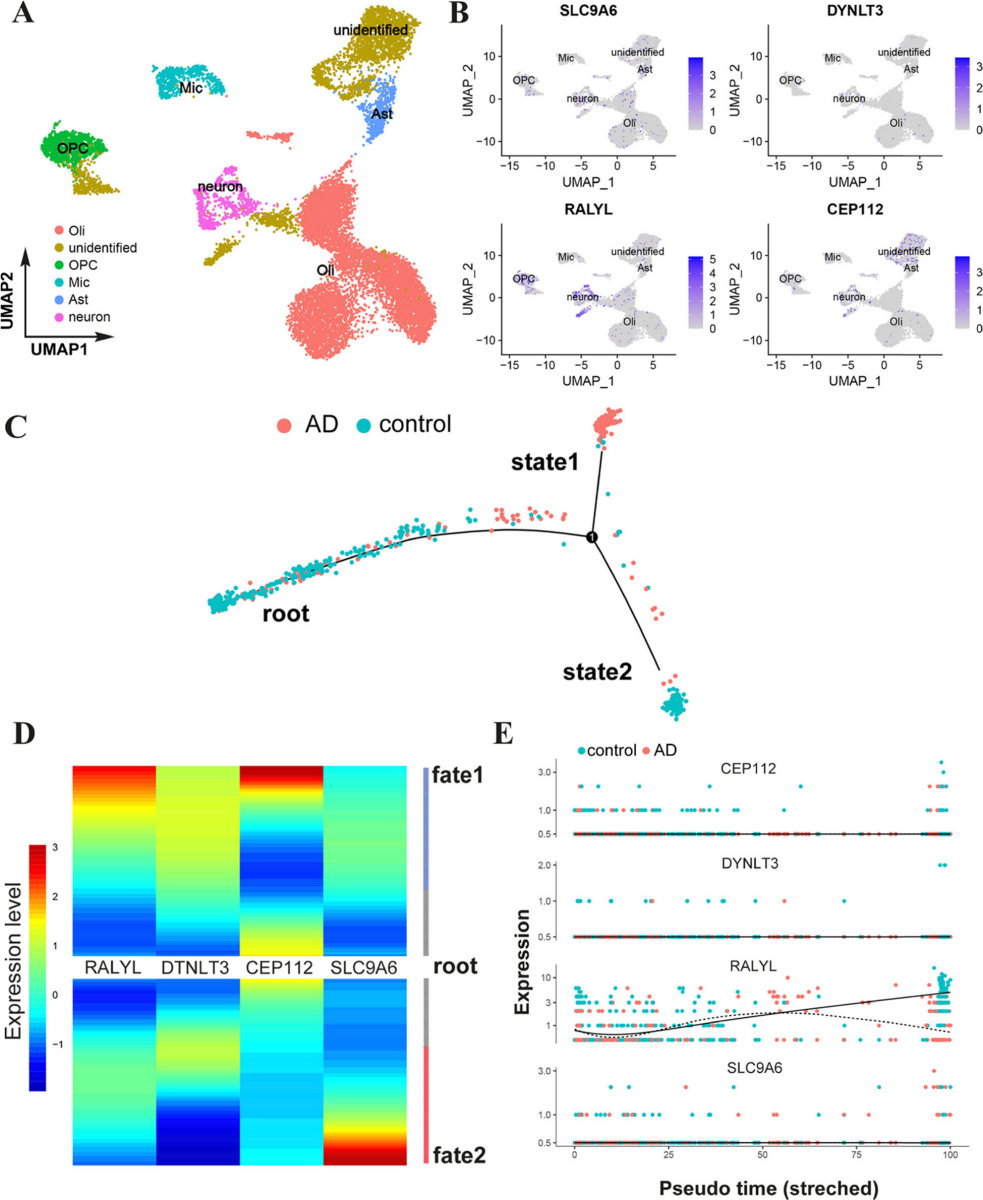
Hub genes’ trajectory inference in single-nuclei sequencing of human AD entorhinal cortex. **a** UMAP visualization showing the clustering of single nuclei, colored by the main five types of brain cells. **b** UMAP visualization showing the location of 4 hub genes within each cell subcluster of brain cell types; *RALYL* is highly expressed in AD neurons. **c** The cell trajectory inference of AD and control in the form of pseudo-time. The trajectory of AD patients is from root to state 1, while control is from root to state 2 over pseudo-time. **d** The hub gene has different expression patterns in state 1 and state 2; *SLC9A6* is highly expressed in state 1 while *RALYL* and *CEP112* are highly expressed in state 2. **e** Pseudo-time projections of hub genes’ drivers in the AD and control subjects, demonstrating the change in relative expression over pseudo-time for the distinct transcriptional states, with each point representing a single cell. Significance based on differential testing by cluster identification used to generate pseudo-time and adjusted for multiple comparisons

Single-nucleus RNA sequencing (snRNA-seq) provides an alternative method for evaluating AD by profiling tens of thousands of individual cells. With this approach, we validated the 6 hub gene expression of module 11 in subjects with a unique cellular-level view of transcriptional alterations. We found cell type-specific and shared gene expression, disease-associated cellular subpopulations, and cell trajectory inference. Exploring the variation of the reserve gene along with the transitions from one cellular state to another enhanced our understanding of whether this gene is involved in the diversion of AD cell fate. We sampled 13,214 cells after quality control and obtained 13,096 cells with a median of 648 detected genes per cell. First, we visualized single-nuclei transcriptomes through uniform manifold approximation and projection (UMAP). The nuclei were separated into 5 clusters, which we mapped to the 5 prior cell types: neurons, astrocytes (Ast), oligodendrocytes (Oil), microglia (Mic), and oligodendrocyte progenitor cells (OPC), based on previously established cell type-specific gene sets (see the “Materials and methods” section) (Fig. 5a). Then, we evaluated the cell-specific expression of the hub gene in UMAP space (Fig. 5b). There were only 4 hub genes (*SLC9A6*, *DYNLT3*, *RALYL*, and *CEP112*) in single-nucleus gene expression atlas. This may be attributed to the fact that this data was retrieved from DroNc-Seq on the 10X platform that highly represents the changes in nuclear transcriptomics [30] but *EBI3* and *TSPYL5* are subcellular localized in the cytoplasm [31, 32]. Notably, *RALYL* transcription in AD neurons was significantly changed, and according to the identified neuron cell clusters, we made the trajectory inference to illustrate the transcript dynamics in AD disease progression.

The neuron cluster contained 702 cells, and using the pseudo-time created by the reverse graph ordering, we produced pseudo-time projections in which we could compare the changes in relative expression over pseudo-time for the distinct transcriptional states. We observed distinct bifurcated architecture of the neuron cell trajectory, implying two different transcriptional states from the root. Most AD subjects were in state 1, while most control subjects were in state 2 (Fig. 5c). There were changes in the expression of hub gene in cell trajectory with different dynamic characteristics. *RALYL* and *CEP112* expression were upregulated from root to stage 2 while *SLC9A6* was upregulated to stage 1 (Fig. 5d). Combined with bifurcated neuron trajectory and transcriptional dynamics, we visualized two kinetic trends for AD and control, one branch located in AD and another in control (Fig. 5e). In summary, among the 6 hub genes for AD reserve, *RALYL* was highly expressed in the neurons of subjects, having similar expression patterns between AD and control at the beginning of AD disease developmental trajectory. However, the expression level decreased since there was a branch point along with the disease progression.

**Fig. 6:**
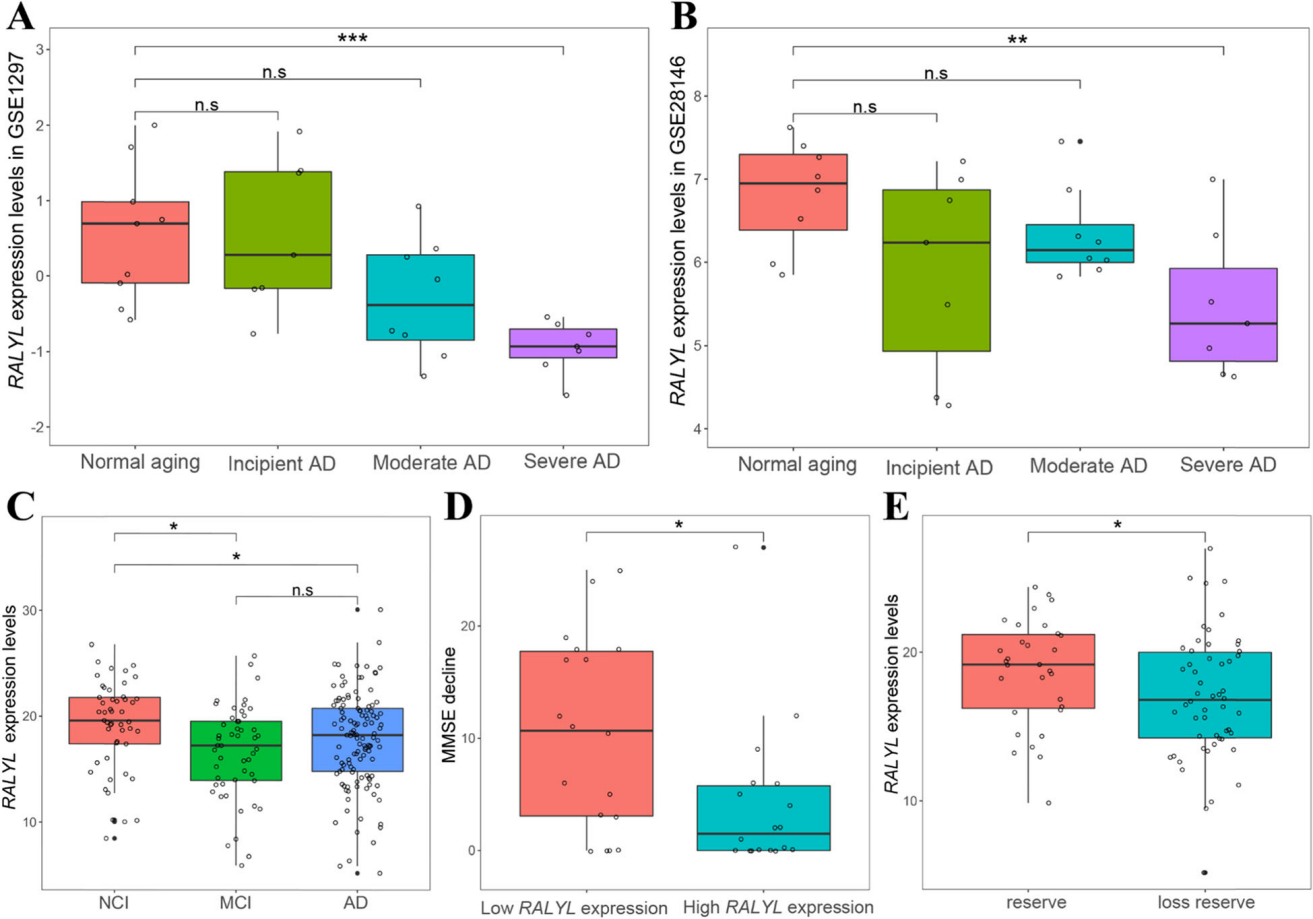
*RALYL* expression dynamic features in AD. **a**, **b** Expression characteristics of *RALYL* in AD disease progression (incipient AD, moderate AD, and severe AD; normal aging as a control group) in AD microarray dataset GSE1297 and GSE28146. *RALYL* decreases during the development of moderate AD to severe AD in GSE1297 (multiple one-way ANOVA, adjusted *p* = 0.0027) and GSE28146 (multiple one-way ANOVA, adjusted *p* = 0.032). **c** *RALYL* expression levels between NCI, MCI, and AD dementia in ROSMAP RNA-seq dataset (multiple one-way ANOVA, adjusted *p* = 0.022). **d** Subjects with reserve have high RALYL expression. Left: MMSE score decline from the first AD diagnoses to last validation from subjects with low *RALYL* expression (fourth quartile of expression levels). Right: MMSE score decline from the first AD diagnoses to last validation from subjects with high *RALYL* expression (first quartile of expression levels) (multiple *t* test, adjusted *p* = 0.019). **e** Specific to AD reserve, subjects with reserve have high expression of *RALYL* than loss reserve subjects (multiple *t* test, adjusted *p* = 0.046)

The dynamic expression feature of *RALYL* demonstrated in other human AD brain transcriptomics datasets causing there no accepted animal model of AD can mimic all aspects of AD currently. In AD disease progression (partition to incipient AD, moderate AD, and severe AD by clinical diagnosis results), *RALYL* was found to maintain the same expression level as in the normal aging group during the conversion of incipient AD to moderate AD, but decreased during the development of moderate AD to severe AD. Normal aging and severe AD group showed significant differences in GSE1297 (multiple one-way ANOVA, adjusted *p* = 0.0027) and GSE28146 (multiple one-way ANOVA, adjusted *p* = 0.032) (Fig. 6a, b). In MCI and AD dementia, subjects had lower *RALYL* expression levels compared to NCI in the ROSMAP RNA-seq dataset (multiple one-way ANOVA, adjusted *p* = 0.022). Cognitive changes showed a high expression of *RALYL* accompanied by an apparent slow cognitive decline in the duplicate MMSE tests (first AD diagnostic MMSE and last valid MMSE) during follow-up (multiple *t* tests, adjusted *p* = 0.019) (Fig. 6d). This explains why subjects with AD reserve had higher *RALYL* expression than those who lost reserve (multiple *t* test, adjusted *p* = 0.046) (Fig. 6e).

## Discussion

AD reserve theory was postulated to provide an interpretation of how a certain degree of neurodegenerative pathology may cause varying degrees of symptoms in different individuals. However, there are several sources of evidence on cognitive resilience and epidemiologic investigation for neurodegenerative diseases and AD dementia. Nevertheless, the mechanisms that underpin the AD reserve protective effects of individual factors remain poorly understood, limiting the development of more preventive, treatment, and translational research. In this study, we adopted a network-based approach and deployed an MPN in identifying specific gene modules associated with AD reserve traits. A key feature of our network-based approach is the identification of unique, direct gene-clinical phenotype-reserve trait relationships, rather than simple protein-protein interaction (PPI) analysis. Furthermore, we validated MPN to ROSMAP cohort, measuring the change in cognition over a long time, the most relevant clinical outcome measure of AD research. A comprehensive characterization of MPN may provide critical insights into the underlying potential AD reserve mechanisms and identify genes that may serve as reserve molecules in AD disease progression for therapeutic intervention.

In this study, using bulk brain transcriptomic data modeling MPN, we obtained 6 hub genes. The single-nucleus transcriptomic data allowed us to identify cellular processes and trajectory inference of these hub genes. However, the single-nucleus data that we used cannot be used to establish MPN because it does not have specific information on the AD phenotype. *RALYL* is the AD reserve correlated with the hub gene for the module and shows different cell trajectories in neurons between AD and control. *RALYL* (RALY RNA Binding Protein-like) is a protein-coding gene. Diseases associated with *RALYL* include renal cell carcinoma and hepatocellular carcinoma [33, 34]. Of note, *RALYL* is an important paralog of this gene. Limited information has been published on its particular role in cognitive performance and AD disease progression. A few studies have, however, documented the potential role of *RALYL* in neurodegenerative disease. For instance, in *RALYL* associated with AD or Parkinson’s disease (PD), human brain samples showed low expression with low variances (AD/control = 0.550; PD/control = 0.660) [35]. This observation was seconded in another research that used substantia nigra tissue samples from PD patients [36]. Moreover, one yeast two-hybrid experiment confirmed the protein interaction between RALYL and LRRK2 [37], while the latter was revealed to play an important role in AD lysosomal dyshomeostasis [38], in synaptic vesicle trafficking [39], and in the regulation of neuronal process morphology in the entire central nervous system [40]. Abundant Aβ pathology has also been found in *LRRK2* mutation carriers and is consistent with comorbid AD pathology [41]. Besides AD and PD, *RALYL* showed single nucleotide polymorphism in amyotrophic lateral sclerosis (ALS), a neurodegenerative disease, following a genome-wide association study (GWAS) [42]. In line with these findings, we now provide evidence that the *RALYL* gene plays a role in AD reserve, and it opens an avenue for investigating the association of *RALYL* with AD.

Our network-based approach analysis not only highlighted the 6 hub genes in module 11 as the target genes that were strongly associated with the AD reserve but also identified some critical genes in hub genes’ expanded subnetwork. As the child nodes of hub genes, these genes are directly regulated, including known AD-related genes (*GABRA1*, *SYN1*, *SORSC3*, *STXBP*) and autism risk gene (*CHD8*). The chromodomain helicase DNA binding protein 8 gene (*CHD8*) provides instructions in generating a protein that regulates gene activity (expression) through chromatin remodeling [43]. The *CHD8* mutation causes autistic-like behaviors [44] and affects the expression of many other genes involved in brain development before birth [45]. Above all, the CHD8 protein and the associated genes regulate the development of neural progenitor cells, giving rise to nerve cells (neurons), and promote growth and division (proliferation), maturation (differentiation), and integration into the neuronal circuitry (migration) [46–49]. These functions are all disordered in AD, which necessitates further exploration of CHD8 function in AD reserve.

In conclusion, AD brain transcriptomics data were clustered into 11 co-expression gene modules by WGCNA. A Bayesian network was established based on these modules and AD reserve related phenotype data. Module 11, which positively regulates neurogenesis, was found to be strongly associated with AD reserve through DAG. Besides, filtration of the hub genes of module 11 by the topological method revealed that of the 6 hub genes in this module, only *RALYL* showed significant transcription changes in AD neurons. These findings imply that the expression of *RALYL* decreases with the gradual progression of AD. Meanwhile, MMSE decline was correlated with RALYL expression. Thus, subjects with AD reserve have significantly higher RALYL expression than those without AD reserve.

## Limitations

Before making a consensus on the 2018 NIA-AA research framework [50], the diagnosis and staging of AD had multiple standards. The clinical traits of AD patients in different databases were not uniform, especially in the diagnosis and staging of subjects. Due to ethical and some other reasons, each database could not disclose the original diagnosis report of the patient, and we were unable to re-stage the subjects according to the latest A/T/N/x standards [50]. Therefore, we could only use the clinical characteristics given by the database. For example, subjects in GSE1297 and GSE28146 were classified as incipient AD, moderate AD, and severe AD, whereas subjects in ROSMAP were classified as NCI, MCI, and AD. These simple subject classification criteria reflect the dynamics of *RALYL* in the disease progression but inaccurately reflect the dynamic relationship between *RALYL* and disease progression related A/T/N/x markers.

It is also important to note the limitations of our approach. The MPN framework increases network accuracy by modeling networks at two resolutions (a zoomed-out module-trait network and a zoomed-in gene network within selected modules). However, higher accuracy was obtained by including only a subset of modules in the inference process, resulting in potential loss of information. Additionally, although *RALYL* has been strictly verified in the human AD brain transcriptomics data, the mechanism by which it plays a specific role in AD reserve and the efficiency or capacity of the reserve should further be explored.

## Conclusion

This study demonstrated the *RALYL* expression dynamics in AD reserve progression and revealed the correlation between *RALYL* expression and cognitive performance through network-based approaches. Our findings provide novel insights into AD reserve and highlight the potential role of *RALYL* in this process.

## Supplementary Information

**Additional file 1: Supplementary Fig. 1 A,** Gene expression dataset GSE1297, including 31 groups of samples were clustered and matched to clinical data. (NA: normal ageing; inAD: incipient AD; moAD: moderate AD; seAD: severe AD) **B,** Analysis of scale-free topology for multiple soft thresholding powers. **Supplement Table. 1.** The main parameters of the DAG nodes.

## Data Availability

The data used during this study are available from the NCBI GEO database (https://www.ncbi.nlm.nih.gov/geo/), Single-cell atlas of the Entorhinal Cortex in Human Alzheimer’s Disease database (adsn.ddnetbio.com), and AMP-AD Knowledge Portal (https://adknowledgeportal.synapse.org/).

## Abbreviations

AD: Alzheimer’s disease
MCI: Mild cognitive impairment
NCI: No cognitively impaired
MPN: Module to the phenotype network
GEO: Gene Expression Omnibus
WGCNA: Weighted gene co-expression network analysis
DAG: Directed acyclic graph
RNA-seq: RNA sequencing
snRNA-seq: Single-nucleus RNA sequencing
NFTs: Neurofibrillary tangles
MMSE: Mini-Mental State Examination
PMI: Postmortem interval
ROSMAP: Religious Orders Study and Memory and Aging Project
TOM: Topology overlay matrix
GO: Gene Ontology
EPC: Edge percolated component
t-SNE: t-distributed Stochastic Neighbor Embedding
UMAP: Uniform manifold approximation and projection
