# Supplementary material for: Exploring the role of RALYL in Alzheimer’s disease reserve by network-based approaches
Source: Alzheimers Res Ther. 2020 Dec 9;12:165. doi: 10.1186/s13195-020-00733-z (PMC7724892; doi:10.1186/s13195-020-00733-z)
Supplement: Supplementary file 1 — Additional file 1: Supplementary Fig. 1 A, Gene expression dataset GSE1297, including 31 groups of samples were clustered and matched to clinical data. (NA: normal ageing; inAD: incipient AD; moAD: moderate AD; seAD: severe AD) B, Analysis of scale-free topology for multiple soft thresholding powers. Supplement Table. 1. The main parameters of the DAG nodes. [file 13195_2020_733_MOESM1_ESM.docx]

**
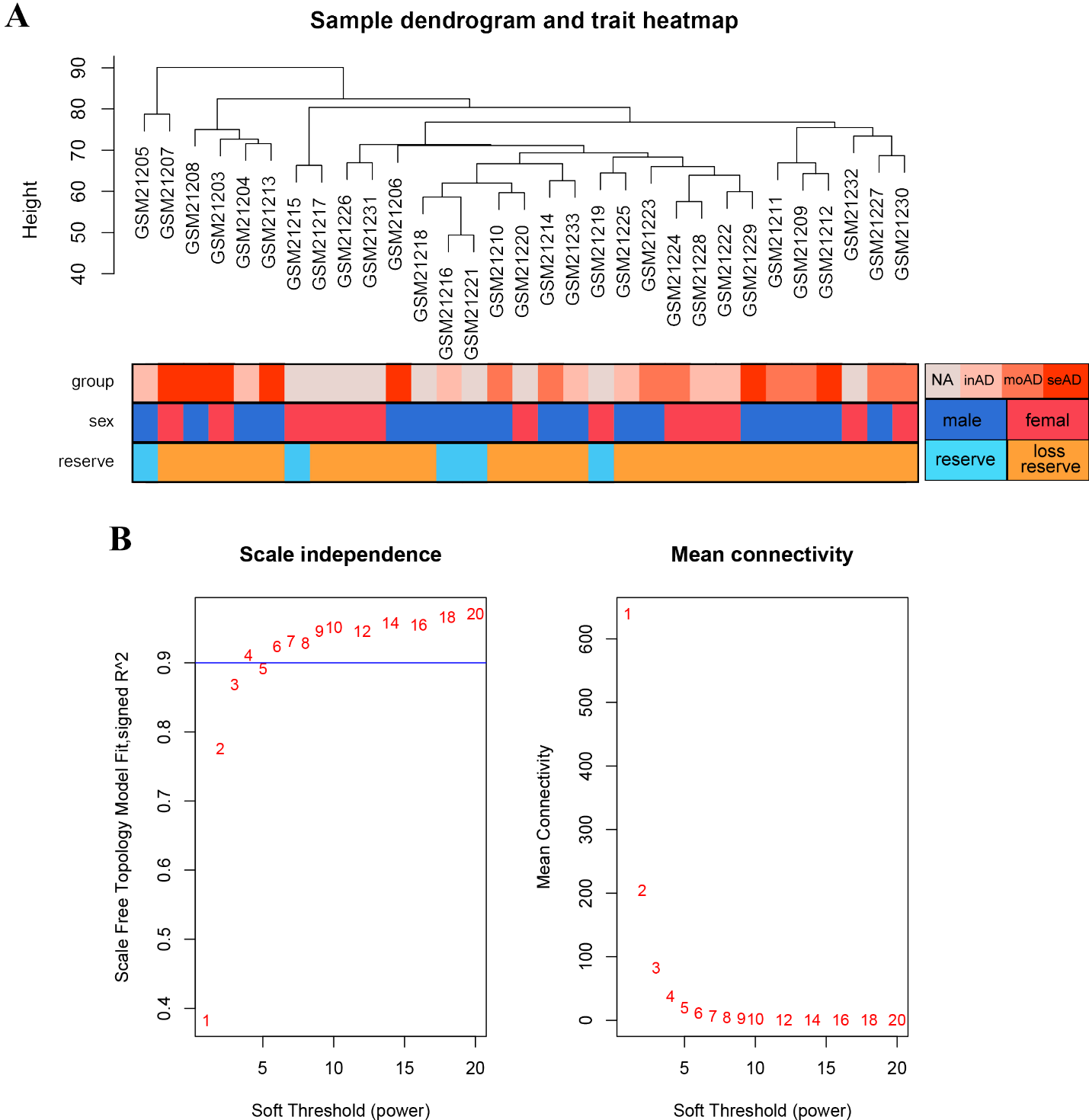
**

**Supplementary Fig. 1 A,** Gene expression dataset GSE1297, including 31 groups of samples were clustered and matched to clinical data. (NA: normal ageing; inAD: incipient AD; moAD: moderate AD; seAD: severe AD) **B,** Analysis of scale-free topology for multiple soft thresholding powers.

| **Supplement Table. 1 The main parameters of the DAG nodes** | | | | | | |
| --- | --- | --- | --- | --- | --- | --- |
| **Gene modules** | **Markov blanket** | **Neighbors nodes** | **Parents nodes** | **Children nodes** | **In degree** | **Out degree** |
| M1 | M2, M3, M4, M6, M9, M11, MMSE, NFTs | M2, M6, M9, MMSE, NFTs | M2, M6, NFTs | M9, MMSE | 3 | 2 |
| M2 | M1, M3, M4, M6, M7, M8, M9, M10, M11, MMSE, NFTs | M1, M3, M4, M7, M9, M10, M11, NFTs | M3, M10, NFTs | M1, M4, M7, M9, M11 | 3 | 5 |
| M3 | M1, M2, M4, M5, M6, M10, M11, MMSE, NFTs | M2, M4, M5, M10, MMSE NFTs | M5, M10, NFTs | M2, M4, MMSE | 3 | 3 |
| M4 | M1, M2, M3, M5, M6, M8, M10, M11, MMSE, NFTs | M2, M3, M8, MMSE, NFTs | M2, M3, NFTs | M8, MMSE | 3 | 2 |
| M5 | M3, M4, M8, M10, NFTs | M3, M8, M10, NFTs | M10 | M1, M3, M6, M7, M8, M9, M11, NFTs | 1 | 3 |
| M6 | M1, M2, M3, M4, M7, M9, M10, M11, MMSE, NFTs, braak, reserve | M1, M7, M10, M11, MMSE, braak | M10, M11 | M1, M7, MMSE, braak | 2 | 4 |
| M7 | M2, M6, M9, MMSE, braak, reserve | M2, M6, M9, braak | M2, M6, M9 | braak | 3 | 1 |
| M8 | M2, M4, M5, M10, M11, NFTs, reserve | M4, M5, M10, M11, NFTs, reserve | M4, M5, M10, NFTs | M11, reserve | 4 | 2 |
| M9 | M1, M2, M6, M7, MMSE | M1, M2, M7, MMSE | M1, M2, MMSE | M7 | 3 | 1 |
| M10 | M2, M3, M4, M5, M6, M8, M11, NFTs | M2, M3, M5, M6, M8, NFTs | -- | M2, M3, M5, M6, M8, NFTs | 0 | 6 |
| M11 | M1, M2, M3, M4, M6, M8, M10, MMSE, NFTs, reserve | M2, M6, M8, MMSE, NFTs, reserve | M2, M8, NFTs | M6, MMSE, reserve | 3 | 3 |

The Markov blanket of a node is the set of nodes consisting of its parents, its children, and any other parents of its children, the Markov blanket renders the node independent of the rest of the network; Neighbourhoods are nodes directly connected to a node, which is a measure of the average density of its neighbourhoods; The number of parents nodes and children nodes is represented by In degree and Out degree.
